# Genome Sequences of *Pseudoalteromonas* Strains ATCC BAA-314, ATCC 70018, and ATCC 70019

**DOI:** 10.1128/genomeA.00390-15

**Published:** 2015-05-07

**Authors:** Scott A. Givan, Ming-Yi Zhou, Karen Bromert, Nathan Bivens, Linda Fleet Chapman

**Affiliations:** a Informatics Research Core Facility, Department of Molecular Microbiology and Immunology, University of Missouri, Columbia, Missouri, USA; b DNA Core Facility, University of Missouri, Columbia, Missouri, USA; c Division of Biological Sciences, University of Missouri, Columbia, Missouri, USA

## Abstract

The assembly and annotation of the draft genome sequences for *Pseudoalteromonas* strains ATCC BAA314, ATCC 700518, and ATCC 700519 reveal candidates for promoting symbiosis between *Pseudoalteromonas* strains and eukaryotes. Groups of genes generally associated with virulence are present in all three strains, suggesting that these bacteria may be pathogenic under specific circumstances.

## GENOME ANNOUNCEMENT

The pseudoalteromonads are of recent interest to ecologists and biochemists due to successful competition in marine habitats and production of pharmacologically active compounds. Knowledge of the genomes of this group also aids in the study of bacteria associated with eukaryotes. Although sometimes isolated as free-living bacteria, pseudoalteromonads have often been isolated as symbionts of algae, fungi, and animals. We have sequenced the genomes of three *Pseudoalteromonas* strains to reveal candidates for promoting interactions between bacterial and eukaryotic cells.

Here, we present the genome sequences of *Pseudoalteromonas flavipulchra* ATCC BAA-314, *Pseudoalteromonas distincta* ATCC 700518, and *Pseudoalteromonas elyakovii* ATCC 700519. ATCC BAA-314 was isolated from seawater off the coast of France (Nice, 43.70N 7.266E), ATCC 700518 was isolated from a marine sponge in the Bering Sea at a depth of 350 Mm, 109 miles east of the Kamchatka Peninsula, and ATCC 700519 was isolated from the coelomic fluid of the Far Eastern mussel *Crenomytilus grayanus* (Vladivostok, Russian Federation, 43.14N 131.89E).

The genomes of the three strains were sequenced using Illumina MiSeq technology. A whole-genome shotgun library was prepared for each strain. The raw reads were trimmed and filtered to remove low-quality bases using the FASTX-Toolkit (http://hannonlab.cshl.edu/fastx_toolkit/index.html). The reads were filtered to remove PhiX using Bowtie, version 0.12.7 (1). SPAdes version 3.1.1 (http://bioinf.spbau.ru/en/spades) was used to assemble ATCC BAA-314. Ray version 2.3.1 (2) was used to assemble ATCC 700518 and ATCC 700519. The assemblies were evaluated and the G+C content determined using Quast version 2.3 (3) and FRC (4). The protein-coding open reading frames were predicted and annotated using the NCBI Prokaryotic Genome Annotation Pipeline. Based on the assembled results, the genome size of ATCC BAA-314 is 4.5 Mb, the G+C content is 43.20%, and the number of scaffolds is 40. The total coverage of the genome is 159×. The genome size of ATCC 700518 is 4.3 Mb, the G+C content is 39.2%, and the number of contigs is 33. The genome coverage is 383.0×. The genome size of ATCC 700519 is 5.45 Mb, with a G+C content of 43.3%, and the number of contigs is 25. The genome coverage is 314.0×.

The annotation results indicate the presence of genes associated with biofilm formation. Groups of genes generally associated with virulence are present in all three strains, suggesting that these bacteria may be pathogenic under specific circumstances.

### Nucleotide sequence accession numbers.

This project has been deposited at the NCBI under the accession numbers JTDZ00000000 (ATCC BAA-314), JWIG00000000 (ATCC 700518), and JWIH00000000 (ATCC 700519). The versions described in this paper are the first versions.
